# Social Comparison and Body Image in Teenage Users of the TikTok App

**DOI:** 10.7759/cureus.48227

**Published:** 2023-11-03

**Authors:** Anas Ibn Auf Abbas Ibn Auf, Yassmeen Hmoud Alblowi, Raghad Oudah Alkhaldi, Salman Anwar Thabet, Ahmed Ali H Alabdali, Fahad Hisham Binshalhoub, Khalid Ali S Alzahrani, Reem Abdulrhman I Alzahrani

**Affiliations:** 1 Department of Psychiatry, Erada and Mental Health Complex, Ministry of Health, Taif, SAU; 2 Department of Psychiatry, Eastern Sudan College for Medical Sciences and Technology, Port Sudan, SDN; 3 Department of Emergency Medicine, King Khalid Hospital, Ministry of Health, Tabuk, SAU; 4 Department of Medicine, Faculty of Medicine, Taif University, Taif, SAU; 5 Department of Psychiatry, Mental Health Hospital, Ministry of Health, Jeddah, SAU; 6 Department of Emergency Medicine, Erada and Mental Health Complex, Ministry of Health, Al-Baha, SAU; 7 Department of Medicine, Faculty of Medicine, Imam Mohammad Ibn Saud Islamic University, Riyadh, SAU; 8 Department of Medicine, Faculty of Medicine, Al-Baha University, Al-Baha, SAU

**Keywords:** saudi arabia, teenagers, body image, social comparison, prevalence, tiktok

## Abstract

Background and aim: The TikTok app is one of the video-based social media platforms that is currently trending and commonly used by teenagers. The data about teenage users of the TikTok app are limited. This study aimed to estimate the prevalence of TikTok app use among teenagers in Saudi Arabia and to investigate the association between TikTok app use and the social comparison and the body image of the study population. Further, to explore the potential associations between the social comparison and the body image of the TikTok app users and their different characteristics.

Methods: This cross-sectional survey study was conducted all over Saudi Arabia. The study included male and female teenagers, aged 12-19 years. The researchers used a structured, self-administered questionnaire to collect data, and it was disseminated using different social media platforms. The social comparison was measured using the questionnaire of the Iowa-Netherlands Comparison Orientation Measure, and the body image was measured using the Measurement of the Body Image Scale. The data were analyzed using the SPSS software version 26 (Chicago, IL: SSPS Inc.). Continuous variables were compared using the Student's t-test, Mann-Whitney U test, or Kruskal-Wallis tests, whereas categorical variables were compared using the Chi-square or Fisher's exact tests as appropriate. Spearman’s rank correlation was applied to investigate the association between body image and social comparison scores among TikTok app users and non-users.

Results: This study included 384 Saudi teenagers. Females (n=260) outnumbered males (n=124) (67.7% and 32.3%, respectively), and the mean of their age was 16.3±2.0 years. The researchers found a high prevalence (343/384, 89.3%) of TikTok app use among the studied teenagers. There was a significantly high median of the social comparison score among the TikTok app users (33.0, IQR: 28.0-38.0) compared to the non-users (28.0, IQR: 26.0-34.0), p=0.005. TikTok app users also showed a significantly lower median body image score (64.0, IQR: 54.0-72.0) in comparison to the non-users (67.0, IQR: 58.0-73.0), p=0.037. The correlation between social comparison and body image among teenagers using TikTok showed a negative relationship, but it was not significant (p=0.110). However, a significant negative weak relationship was found between body image and comparison of abilities (r_s_ coefficient=-0.113, p=0.037).

Conclusions: The findings of the current study indicate a high prevalence of TikTok use among teenagers in Saudi Arabia. The use of TikTok was associated with a high social comparison and a negative body image, with more than half of the users expressing a negative body image. Comparison of abilities was associated with an increased level of body dissatisfaction. These findings emphasize the need for the development and implementation of public health policies and awareness programs to promote body acceptance, especially for young people across the country. Future studies should focus on the long-term impact of TikTok use on psychological health.

## Introduction

Social media use either for entertainment or communication purposes has become an essential aspect of people’s life [1]. In Saudi Arabia, social media usage experienced a rapid rise with 28.81 million people (79.30% of the population) using social media in 2023 [2]. This figure is much higher among Saudi young people, with 98.43% of them using social networking sites [3].

TikTok app is one of the video-based social media platforms that is currently trending and commonly used by teenagers. Some videos with adversely related content are detrimental to adolescents and impact their body image [4]. The problem is greater among adolescents because many of them are not yet mature enough to differentiate between unrealistic videos and reality [5].

Body image denotes the cognitive attitudes and evaluations perceived by individuals about themselves, and relates to physical appearance including body size and shape [6]. Teenagers have the concept of an ideal body image and they interact with the social environment in the form of social comparisons with other people [7].

Pitron et al. established that exploring the relationship between TikTok and perceived body image is essential because negative body image can often lead to body dissatisfaction, low self-esteem, dieting, and eating disorders [8].

Numerous studies have been conducted about the association between using smartphones and social media networks and social comparison, body image, and behavioral disorders in Saudi Arabia, but the data about teenage users of the TikTok app are limited [9-12]. Therefore, this study aimed to estimate the prevalence of TikTok app use among teenagers in Saudi Arabia and to investigate the association between TikTok app use and the social comparison and the body image of the study population. Further, this study aimed to explore the potential associations between the social comparison and the body image of TikTok app users and their different characteristics.

## Materials and methods

Ethical considerations

All participants were informed about the study objectives, methodology, risks, and benefits. Subjects, who agreed to fill out the questionnaire, implied that they agreed to participate in the study. The study was conducted after obtaining ethical approval from the Research and Studies Department of the Directorate of Health Affairs, Taif, Saudi Arabia (approval number: 805, date: May 15, 2023).

Study design, setting, and date

This was a cross-sectional survey study that was conducted all over Saudi Arabia between June 2023 and July 2023.

Sample size calculation and sampling technique

The sample size was estimated with an online sample size calculator (Raosoft; Seattle, WA: Raosoft Inc. http://www.raosoft.com/samplesize.html) using a margin of error of 5% and a confidence interval of 95%, assuming an average response for most of the questions of 50%, and depending on an average population size of nearly 900,000 teenage population in Saudi Arabia [13]. The required sample size was 384. The researchers used the non-probability convenience sampling technique to collect the predetermined response rate.

Eligibility criteria

The study involved the general population of Saudi Arabia. The study included male and female teenagers aged 12-19 years. The researchers excluded responses from younger (less than 12 years old) and older (over 19 years old) participants and those with incomplete data.

Data collection tool

The researchers used a structured, self-administered questionnaire to collect data. The questionnaire was divided into four parts. The first part collected the participants’ characteristics. The second, third, and fourth parts focused on the participants’ TikTok app use, the social comparisons scale, and the body image scale, respectively.

The social comparison was measured using the questionnaire of the Iowa-Netherlands Comparison Orientation Measure proposed by Gibbons and Buunk [14]. It included 11 items, where people were given statements about their self-comparisons with others, to which they can respond on a five-point scale ranging from “strongly disagree” to “strongly agree.” Its validity and reliability have been proven based on a wide range of empirical tests. It differentiated between two dimensions of social comparisons that were distinct in their underlying nature: (a) comparisons of abilities referring to the question “How am I doing?” (items 1-6) and (b) comparisons of opinions referring to the question “What shall I feel/think?” (items 7-11). Each dimension included a reverse-coded item (items 5 and 11) as a control for acquiescence biases.

The body image was measured using the Body Image Scale, which was designed, translated to Arabic, and validated by Abdul-Naby [15]. It was also used by other studies, such as Keshk et al. [16]. It consisted of 27 items and fell into two dimensions: (a) the individual’s perception of his body, which is positive or negative, and (b) the individual’s perception of his body through the opinions of others, such as family, friends, and colleagues. The answer fell on three levels: “yes,” “sometimes,” and “no”. It evaluated “yes” with three degrees, “sometimes” with two degrees, and “no” with one degree, in the positive expressions (1, 2, 7, 10, 11, 16, 22, 24, 25, 27). The scores were reversed in the negative expressions (3, 4, 5, 6, 8, 9, 12, 13, 14, 15, 17, 18, 19, 20, 21, 23, 26).

Data collection procedure

An online structured questionnaire was designed on “Google Forms” and presented to the participants to collect data. It was disseminated using different social media platforms, such as Facebook, Twitter, Instagram, and WhatsApp. The participants were informed about the study objectives, methodology, risks, and benefits, and they were asked to give informed consent to participate in the study before starting to fill in the questionnaire.

Statistical analysis

The tabulation and analysis of data were carried out using the SPSS software version 26 (Chicago, IL: SSPS Inc.). Continuous variables were reported as mean±standard deviation (SD) or median with interquartile range (25th-75th percentiles) depending on the normality of the distribution. The parametric Student’s t-test and the non-parametric Mann-Whitney U and Kruskal-Wallis tests were applied for comparison between groups. Categorical variables were reported as frequencies and percentages and were compared using Chi-square or Fisher’s exact tests as appropriate. Spearman's rank correlation was applied to investigate the association between body image and social comparison scores among TikTok app users and non-users. A p-value <0.05 is considered statistically significant.

## Results

This study included 384 Saudi teenagers. Females (n=260) outnumbered males (n=124) (67.7% and 32.3%, respectively), and the mean of their age was 16.3±2.0 years. The study involved all regions of Saudi Arabia, but the Southern (n=140, 36.5%) and the Western (n=114, 29.7%), followed by the Eastern (n=71, 18.5%) regions were the most represented. The secondary (n=183) and middle (n=113) schools (47.7% and 29.4%, respectively) were the most frequent education levels of the participants. More than half (n=215, 56.0%) reported a family monthly income of more than 10,000 Saudi Riyals (Table 1).

**Table 1:** Sociodemographic characteristics of the study participants (N=384). N: number; SD: standard deviation

Variables		N=384	%
Gender	Female	260	67.7%
	Male	124	32.3%
Age (years)	Range	12.0-19.0	
	Mean±SD	16.3±2.0	
Residence	Southern region	140	36.5%
	Western region	114	29.7%
	Eastern region	71	18.5%
	Central region	33	8.6%
	Northern region	26	6.8%
Education level	Elementary school	8	2.1%
	Middle school	113	29.4%
	Secondary school	183	47.7%
	University	80	20.8%
Family monthly income (Saudi Riyal)	Up to 2,000	15	3.9%
	>2,000 to 5,000	51	13.3%
	>5,000 to 10,000	103	26.8%
	>10,000	215	56.0%

**Figure 1:**
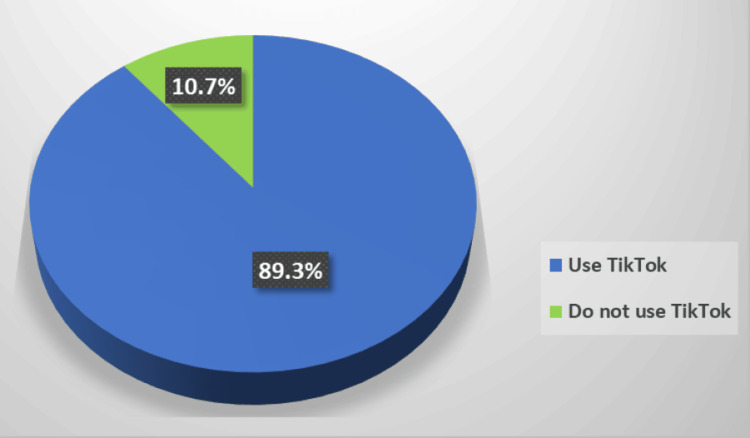
Prevalence of TikTok app use among the studied teenagers.

**Figure 2:**
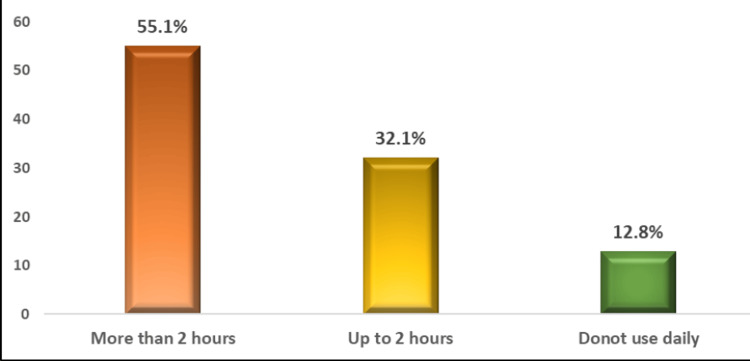
Time spent daily on the TikTok app.

We found a high prevalence (343/384, 89.3%) of TikTok app use among the studied teenagers (Figure 1). The daily use of the TikTok app was either for more than two hours (189/343, 55.1%) or up to two hours (110/343, 32.1%) while a few percentages (44/343, 12.8%) were not using it daily (Figure 2).

The value of the total social comparison score ranged from 13 (minimum) to 51 (maximum), with a median score of 33. Scores higher than 33 indicated high rates of social comparison concerning various life situations, while scores lower than 33 pointed to low rates of social comparison. The value of scores on the Body Image Scale ranged from 27 (minimum) to 80 (maximum). The median was calculated and found to be 64. Scores higher than 64 indicated a positive body image and the individual’s real and clear perception of his body image and his satisfaction with it, and scores of 64 or lower indicated a negative body image and the individual’s wrong perception of his body image and his dissatisfaction with it. The results showed a significantly high median of the social comparison score among the TikTok app users (33.0, IQR: 28.0-38.0) compared to the non-users (28.0, IQR: 26.0-34.0), p=0.005. The comparison of abilities scores also showed a significantly higher median among the TikTok app users than in the non-users (p=0.002). Alternatively, there were no significant differences between both groups regarding the comparison of opinions score (p=0.208). Furthermore, the TikTok app users showed a significantly lower median body image score (64.0, IQR: 54.0-72.0) in comparison to the non-users (67.0, IQR: 58.0-73.0), p=0.037 (Table 2).

**Table 2:** Comparison between the TikTok app users and non-users regarding the social comparison and the Body Image Scores. *P-value <0.05 is considered statistically significant. N: number; IQR: interquartile range

Variables		TikTok app users, N=343	TikTok app non-users, N=41	Total, N=384	p-Value
Total social comparison score	Median	33.0	28.0	33.0	0.005*
	IQR	28.0-38.0	26.0-34.0	27.0-38.0	
Comparison of abilities score	Median	17.0	14.0	17.0	0.002*
	IQR	14.0-21.0	13.0-16.0	14.0-20.5	
Comparison of opinions score	Median	16.0	15.0	16.0	0.208
	IQR	13.0-19.0	13.0-18.0	13.0-18.0	
Body image score	Median	64.0	67.0	64.0	0.037*
	IQR	54.0-72.0	58.0-73.0	54.0-72.0	

The correlation between social comparison and body image among teenagers using TikTok showed a non-significant (p=0.110) negative relationship, but a significant (p=0.037) negative weak relationship between body image and comparison of abilities (r_s_ coefficient=-0.113) (Table 3).

**Table 3:** Correlations between the body image and the social comparison scores among users and non-users of TikTok app. *P-value <0.05 is considered statistically significant. N: number; r_s_ coefficient: Spearman’s rank correlation

Variables	TikTok app users, N=343	
	Body image score	
	r_s_ coefficient	p-Value
Total social comparison score	-0.086	0.110
Comparison of abilities score	-0.113	0.037*
Comparison of opinions score	-0.013	0.811

About two-thirds (232/343, 67.6%) of the teenagers using the TikTok app were females, with a mean age of 16.4±2.0 years. Most of them were in the middle (98/343, 28.6%) and secondary schools (166/343, 48.4%), and more than half (192/343, 56.0%) have a family monthly income greater than 10,000 Saudi Riyals. Furthermore, there was a significant association between gender and the perceived body image by TikTok app users (p=0.040). A higher percentage of individuals having a negative body image were females (119/189, 63.0%). The users having a negative body image were significantly younger than those having a positive body image (p=0.003). The mean age was 6.1±2.0 and 16.7±1.81, respectively. The body image of the users also showed a significant relationship with the education levels (p=0.005). A high proportion of the negative body image users were middle and secondary school students. Significantly greater percentages of the teenagers with a negative body image were residing in the Western (67/189, 35.4%) and the Southern (57/189, 30.2%) regions of Saudi Arabia (p=0.012) and having a family monthly income greater than 10,000 Saudi Riyals (98/189, 51.9%), p=0.018 (Table 4).

**Table 4:** Associations between sociodemographic characteristics and the body image type among TikTok app users (N=343). *P-value <0.05 is considered statistically significant. N: number; SD: standard deviation

Variables		Negative body image, N=189 (55.1%)		Positive body image, N=154 (44.9%)		Total TikTok users, N=343		p-Value
Gender	Female	119	63.0%	113	73.4%	232	67.6%	0.040*
	Male	70	37.0%	41	26.6%	111	32.4%	
Age (years)	Mean±SD	6.1±2.0		16.7±1.81		16.4±2.0		0.003*
Region	Southern	57	30.2%	68	44.2%	125	36.4%	0.012*
	Northern	14	7.4%	8	5.2%	22	6.4%	
	Eastern	32	16.9%	34	22.1%	66	19.2%	
	Western	67	35.4%	34	22.1%	101	29.4%	
	Central	19	10.1%	10	6.5%	29	8.5%	
Education level	Elementary school	5	2.6%	2	1.3%	7	2.0%	0.005*
	Middle school	67	35.4%	31	20.1%	98	28.6%	
	Secondary school	77	40.7%	89	57.8%	166	48.4%	
	University	40	21.2%	32	20.8%	72	21.0%	
Family monthly income (Saudi Riyal)	Up to 2,000	12	6.3%	1	0.6%	13	3.8%	0.018*
	>2,000 to 5,000	30	15.9%	17	11.0%	47	13.7%	
	>5,000 to 10,000	49	25.9%	42	27.3%	91	26.5%	
	>10,000	98	51.9%	94	61.0%	192	56.0%	

The medians of the social comparison score were comparable in both females (33.0) and males (35.0), with no significant difference (p=0.125). Different regions of Saudi Arabia also did not show significant differences regarding the median social comparison score (p=0.750). There was no significant association between the education level and the social comparison score (p=0.260). The median score was non-significantly slightly higher in the elementary and middle schools (37.0 and 35.0, respectively) than in the middle and university education (median=33.0 each). Furthermore, there was no significant relationship between the family income and the social comparison score (p=0.771) (Table 5).

**Table 5:** Comparison of the social comparison score among different sociodemographic characteristics of TikTok app users (N=343). N: number; IQR: interquartile range

Variables		Social comparison score		p-Value
		Median	IQR	
Gender	Female	33.0	28.0-38.0	0.125
	Male	35.0	28.0-40.0	
Region	Southern	33.0	27.0-39.0	0.750
	Northern	33.0	23.0-39.0	
	Eastern	34.0	28.0-39.0	
	Western	34.0	29.0-38.0	
	Central	33.0	28.0-38.0	
Education level	Elementary school	37.0	26.0-39.0	0.260
	Middle school	35.0	30.0-39.0	
	Secondary school	33.0	26.0-38.0	
	University	33.0	28.0-38.0	
Family monthly income (Saudi Riyal)	Up to 2,000	31.0	27.0-38.0	0.771
	>2,000 to 5,000	32.0	26.0-38.0	
	>5,000 to 10,000	33.0	28.0-39.0	
	>10,000	33.5	28.0-39.0	

## Discussion

Social media usage provides several opportunities for youth, but it has become a serious public health concern due to its adverse psychological effects [17]. The popular TikTok social media app is used easily where the users can watch, share, or create their content [18]. The inexperienced nature of the young users might affect their sense of self and others, and this critical issue requires a thorough investigation [19].

In the present study, the researchers explored a high prevalence (343/384, 89.3%) of TikTok app use among Saudi teenagers, with more common use among females (232/343, 67.6%) and students in the middle (98/343, 28.6%) and secondary schools (166/343, 48.4%). These findings are in line with previous studies in Saudi Arabia which reported a 55.2% (180/326) prevalence of social media addiction among medical students at King Khalid University [20], and a 50.1% (154/307) prevalence of moderate social media addiction among Princess Nourah University female students [21]. In China, more than half of the internet users are using TikTok daily because they think that it is a fashionable lifestyle [22]. Lu et al. investigated the motivations for TikTok use and revealed that the engaged users were satisfied with the content of the videos and thought that many videos were valuable [23]. Alternatively, the non-users described that a lot of videos are of low quality and value. Moreover, they found that recommendation algorithms of TikTok play an important role in engaging users.

Another aspect of using TikTok is the uncontrollable cravings to use the platform and spending much time using it, which impairs the users’ psychosocial health and well-being [20]. In this context, more than half (189/343, 55.1%) of the studied teenagers reported using it for more than two hours daily and only a few percentages (44/343, 12.8%) were not using the app daily. The time spent on social media lowers the amount of time available for other activities such as sports, community service, and family time.

The present study revealed that the use of TikTok app was associated with a high social comparison score, particularly the comparison of abilities which was the most prominent among the TikTok app users compared to the non-users. Teenagers pay more attention to their bodies and consider physical appearance an important factor in social relationships as it is most easily seen by others [24]. This concept is the basis of the phenomenon of social comparisons recorded among TikTok users. They compare themselves against people who upload video content on TikTok which lowers their level of confidence [25].

In the present study, TikTok app use was also associated with a low body image score, and more than half (189/ 343, 55.1%) of the users perceived a negative body image about themselves. Negative body image indicates that the users perceive their features and body size as distorted. The worldwide increased interest in body image perception is due to the serious consequences of distorted or negative body image on physical, psychological, and behavioral well-being. Unhealthy attitudes and practices regarding eating behaviors such as strict dieting and excessive weight control, in addition to low self-esteem, and life dissatisfaction have been recorded [16,26]. A study from Brazil found a significant association between mass media use and increased body dissatisfaction among adolescents [27]. Alternatively, previous studies in Saudi Arabia revealed a low level of body dissatisfaction among college students using social media [28] and an absence of a significant association between social media and body image [21]. Xu et al. suggested that the negative influence of TikTok use on body image is related to the content consumed by the users [29]. Individuals who cannot reach the ideal body sizes represented in the media may develop a negative body image. Likewise, Ayran et al. reported a negative correlation between the level of internet addiction and satisfaction with body image [30].

In this study, there was a significant negative correlation between the comparison of abilities score and body image; the higher the adolescents make abilities comparisons, the lower their body image and vice versa. Rousseau et al. showed an increased level of body dissatisfaction among individuals who compare themselves with the posts they read on Facebook [31]. Another study from Canada also showed increased concerns of young females who compare their body appearance with peers on social media [32]. Nevertheless, Rahmadiansyah et al. found a significant positive relationship between social comparisons and body image in teenagers using TikTok [33]. Pan et al. reported that passive and active TikTok uses were negatively and positively associated with participants' appearance- and weight-esteem, respectively, which reflects the complicated impact of TikTok use on body image [34].

The current survey revealed a significant association between gender and the perceived body image by TikTok app users. Females have suffered from negative body image to a greater degree than males. This agrees with Hogue et al. who stated that females are more concerned with their looks than males and that males are more likely to use social media to find friends [32]. However, it has been suggested that both genders suffer body dissatisfaction, but they express it in a different way which might be due to gender variations in cultural norms regarding the body. Body dissatisfaction in females is mainly related to body weight with striving to be thinner while males tend to report greater dissatisfaction with muscularity and lean body mass [35].

The TikTok app users also showed a significant association between the perceived body image and other sociodemographic characteristics such as age, education level, residence, and family income. Greater percentages of the users who perceived negative body image were young, middle, and secondary school students, residing in the Western and the Southern regions of Saudi Arabia, and having a family monthly income greater than 10,000 Saudi Riyals. This information is helpful for decision-makers, health providers, and also the researchers.

Strengths and limitations

To the best of the researchers' knowledge, this study is the first in Saudi Arabia to estimate the prevalence of TikTok among teenagers and to investigate the association between TikTok use and social comparisons and negative body image. However, it is limited by its cross-sectional design which could not guarantee a cause-effect relationship, and the convenience sampling that has greatly restricts the extrapolation of the results. In addition, using an online, anonymously filled-in questionnaire on self-reported data might cause a data collection error as well as a selection bias.

## Conclusions

The findings of the current study indicate a high prevalence of TikTok use among teenagers in Saudi Arabia, with more common use among females and middle and secondary school students. The TikTok users showed a high social comparison and a negative body image, with more than half of them expressing a negative body image. Females and young individuals have suffered from negative body image to a greater degree than males. Furthermore, there was an increased level of body dissatisfaction among adolescents who compared their abilities with others. Future studies should focus on the long-term impact of TikTok use on psychological health. These findings highlight the need to develop and implement social media-related public health policies and awareness programs for promoting body acceptance, particularly targeting the youth across the country.
